# Playing the safe card or playing the race card? Comparison of attitudes towards interracial marriages with non-white migrants and transnational adoptees in Sweden

**DOI:** 10.1186/s40878-018-0074-6

**Published:** 2018-05-17

**Authors:** Sayaka Osanami Törngren

**Affiliations:** Malmö Institute for Studies of Migration Diversity and Welfare, Malmö University, 205 06 Malmö, Sweden

**Keywords:** Attitudes, Interracial marriage, Transnational adoptees, Sweden, Mixed methods, Race, Visible differences

## Abstract

This article compares the attitudes of white Swedes towards interracial marriages with someone of non-white migrant origin and a non-white transnational adoptee. The analysis is based on a postal survey and follow-up interviews conducted in Malmö, Sweden. Survey results show that transnational adoptees are not preferred as marriage partners by white Swedes to the same extent as white Swedes. Moreover, the differences in attitudes towards marriages with migrants and non-white adoptees are not statistically significant. Interviewees utilized the notion of cultural differences to explain the attitudes towards intermarriages with migrants. However, this was highly contested when talking about the attitudes towards non-white transnational adoptees. These results show how race and visible differences play a role in attitudes toward interracial marriages in Sweden.

## Introduction

Post-war Sweden has been a country of immigration; people of diverse ethnic and racial backgrounds have become an undeniable part of present Swedish society. Today 16% of some 10 million persons living in Sweden are foreign-born (Statistics Sweden, 2017). Due to the rising number of migrants and migrant descents born in Sweden, interracial contact has become inevitable in society and in people’s everyday lives, particularly in Sweden’s main cities. As a consequence, choosing a partner from a different ethnic and racial background than oneself is now more prevalent and conventional. Root writes, “[i]nterracial relationships, including interracial marriage, are natural consequences of increased social interaction between races” (Rooth, 2001, p. 3). Marriage is one of the most personal and intimate social relationships that individuals enter into. This relationship is unlike other types of social relationships, for example in the workplace or in public spaces, where the choice of not interacting across the ethnic and racial boundaries is limited. It is one of the few relationships where “the member of the ethnic group may if he wishes follow a path which never takes him across the boundaries of his ethnic structural network” (Gordon 1961, p. 280). Therefore, as Yancey and Lewis (2009) suggests, people rejecting or opposing interracial relationships would, with or without intention, legitimize the boundary of us and them, thereby legitimizing racial discrimination and prejudice. Research on intermarriage is still scarce in the Nordic context, particularly studies on attitudes towards intermarriages that look at the preferences of potential marriage partners.

This article examines the attitudes of white Swedes toward interracial marriage^1^ in Sweden. It is based on 420 survey responses and 28 interviews with people of white Swedish origins^2^ residing in Malmö, Sweden. Because statistics in Sweden show that persons of Swedish origin mostly intermarry with persons of European origin (e.g. Crester, 1999; Niedomysl, Östh, & Van Ham, 2010; Stenflo, 2001), it is of interest to focus on what their attitudes are towards marrying somebody of non-European origins. Even though other factors such as ethnicity, religion or language may play a role, this article will focus solely on how ideas of race invoked by visible differences form the perception of culture and cultural differences of the imagined partner, which in turn may play a role in marriage preferences. In particular, this article focuses on the question of how race and visible differences of the imagined partner plays a role by comparing attitudes towards having a spouse of non-white migrant origin or transracial adoptee, i.e. persons adopted from a non-European country to a Swedish family as a child.

Previous studies in Sweden have argued that visible differences, such as skin color and other features, are interpreted as cultural differences (e.g. Pred, 2000). By comparing attitudes towards partners of migrant origin and non-white transnational adoptees, the role of the visible differences in the perception of culture is highlighted. Transnational adoptees who grow up in Sweden as culturally Swedish have traditionally not been included in the field of migration studies. Adoptees differ considerably from other migrant groups in Sweden in their language ability, cultural background and social networks. They generally grow up in a Swedish family, with a Swedish-sounding name, and with Swedish as their mother tongue. Therefore, observing attitudes towards marriages with such adoptees and comparing them with migrants of the same region of origin enables the study to ask the following two questions: How can attitudes towards interracial marriages be explained by cultural preferences? And how can such attitudes be explained by visible differences and perceptions of race?

### Previous studies on interracial marriage in Sweden

Intermarriage has been widely studied in the North-American and British context. The topic has gained more attention in the Nordic context in recent years, although, as Olofsson (2007) indicates, it has not yet been widely researched or studied in Sweden. Studies have mapped the number of intermarriages and analyzed who marries whom (e.g. Dribe & Lundh, 2011, Niedomysl, et al., 2010; Statistics Sweden, 2010). However, as Swedish statistics do not provide any information about the ethnic or racial backgrounds of individuals, intermarriage is usually defined as a union between individuals of different nationality or country of birth. Moreover, the studies employ different definitions of what constitutes intermarriage. As a result, different studies refer to the majority and minority populations in different ways. For example, they use concepts such as foreign-born, Swedish-born or first/s generation and ethnic Swedes, based on one’s country of birth and that of one’s parents. Due to the ambiguity of the categories, the actual number of intermarriages in Sweden remains unclear. An important finding from the Swedish studies is that statistics show an increase in intermarriages in recent years. Crester’s study (1999) defines intermarriage as a marriage between a Swedish citizen and non-Swedish citizen, and it shows a 50% increase in the rate of intermarriage from the 1970s to the 1990s. The latest report from Statistics Sweden (2010) defines intermarriage through the country of birth of one’s parents. Further, it reports that from 2004 to 2008, a total of 9% of all established marriages were intermarriages between Swedish-born persons with both parents born in Sweden and foreign-born persons.

Some opinion surveys include questions that specifically address attitudes towards intermarriage, such as Lange and Westin’s study (1997), Society Opinion Media surveys (Demker, 2005), and a report by the former Swedish Integration Board, Integration Barometer (Integrationsverket, 2006, 2007). The results of these surveys mostly show positive attitudes towards building a family across racial and ethnic lines. Contrary to the Society Opinion Media survey and Integration Barometer’s results, both of which indicate that very few individuals oppose intermarriage, some ethnographic studies reveal that people who are actually involved in interracial relationships often meet resistance from society (e.g. Begovic, 2003; Gerholm, 2000, 2003).

None of the above studies in Sweden include non-white transnational adoptees; moreover, the role of race and visible differences are not specifically raised. Hübinette and Tigervall (2008, 2009) state that Swedish migration scholars have not included adoptees in the field because of their cultural belonging in Sweden. However, recent studies on adoptees and their experiences of discrimination and racism highlight the need to question their (in)visibility in debates on both; they also highlight the importance of including them in the field of international migration and ethnic relations (e.g. Hübinette & Tigervall, 2009; Lundström, 2007; Signell & Lindblad, 2008; Signell, 2006). Though adoptees are culturally Swedish, they share the same racial background as migrants. Consequently, comparing attitudes towards non-white migrants and transnational adoptees will highlight whether and when culture and race matter in Sweden.

## Theoretical approach

### Perception of culture based on visible differences

Cantle asserts that the term “culture” is often used as an overarching concept that describes various differences. Even though culture is defined differently in academia by various scholars, it is commonly defined in terms of shared values and a way of life specific to a group of people. Cantle states that the concept of culture as a reference to difference is vague and problematic as culture is “inevitably subjective, defined by the individuals and groups themselves – and by the perceptions of others” (2005, p. 85). It is, therefore, difficult to define what culture is and to draw boundaries accordingly. It is also important to make a distinction between how culture is defined and what the perception of culture is. Barth argues that culture is often used in public to refer “selectively for that which seems most salient to the outsider, namely difference” (1995, p. 65). The concept of culture is applied to individuals or a group of people who are perceived to be different from us. It is something that is “exotic” and special to the other; the concept of culture is increasingly used as “identity” (Barth, 1995, p. 65). Quoting Banton, Norman maintains:

‘social differences are often taken for granted when they seem to have a physical basis’ (1988:8) and people will nonetheless tend to classify immigrants, refugees, and foreigners into different cultural categories according to cultural perceptions of phenotypical variation or physical appearance, such as ‘Arab-looking, black, Indian, Korean’. (Norman, 2004, p. 210)

This practice of perceiving cultural differences through phenotypical variations can be seen in the Swedish context as well. For example, Pred argues that visible differences like skin color or other bodily markers are interpreted as cultural differences in Swedish society (2000). The perception of culture depends on identifying differences that are visible. Moreover, Mattsson argues that the perception of Swedishness is based on visibility, whether you “look like a Swede” and “have a Swedish appearance,” which has a direct connotation to whiteness and Europeanness (2005, p. 150).

This article treats visible differences such as skin and hair color as markers used to categorize individuals into socially-constructed races. Race is something that exists, not as a biological reality but as a social reality. I argue that race is a social reality because the lives of the people who are categorized as different due to their visible differences experience discrimination and racism (e.g. Bonilla-Silva, 2010; Omi & Winant, 1994). Stating that race is a social reality does not mean that I treat race as something essential and static: ideas of race and racial categories change over time, place and context.

### Talking about race in Sweden

The concept of race is not widely applied as a theoretical concept in Sweden today. The word race is most often replaced with words such as “origin” or “ethnic groups” (Wigerfelt, 2004, p. 26). Although many different definitions of ethnicity can be found, they all feature a common denominator: the notion of a shared culture (e.g., Cornell & Hartmann, 2007; Fenton, 2003). Many researchers conflate the meaning of race and ethnicity; however, the two are distinct in that race is visible phenotypically, whereas ethnicity is not always so. Race is often an ascribed and assigned category, while ethnicity, when compared to race, is often acquired and self-claimed by the individuals in the group.

Colorblindness – “a mode of thinking about race organized around an effort to not ‘see,’ or at any rate not to acknowledge, race differences” (Frankenberg, 1993, p. 142) – can be observed in Sweden.^3^ Differences between the majority and minority are discussed by using ambiguous terms such as “immigrants” and “country of birth” (Brekke & Brochgrevink, 2007). Despite this, previous studies on racism and discrimination in Sweden highlight how visible differences, such as skin color, play a role in Swedish society (e.g. Kalonaityte, Kawesa, & Tedros, 2007; Khosravi, 2006; Lundström, 2007; Sawyer, 2000). As seen in the debate between Rabo and Andreassen (2014), there are “national and generational” differences in the discussions of whether we should or should not talk about race in the Nordic context. Failing to talk about race and the role of visible differences is to ignore the effects that the visible differences have on some groups of people and their social lives. In this article, I intend to contribute to the discussion on the significance and roles of race and the visible differences in Swedish society by comparing attitudes towards interracial marriages with transnational adoptees and migrants of the same origin.

### Operationalizing race and constructing racial groups

Ratcliffe explains the struggle experienced by scholars who are skeptical about the use of the term race; he states, “[t]here is no ideologically or methodologically neutral ways of expressing differences between peoples of differing heritages” (2004, p. 24). Since ethnicity is often used as a way to categorize different migrant groups in Sweden, the racial groups used in this study have been constructed only for the purpose of this study. The groups should be understood as “ideal-types”, which are “a rational construction used to make sense of and explain an irrational reality” (Daynes & Lee, 2008, p. 94) and are used as a methodological convenience (Weber, 1978). A construction is simply a way of understanding social reality. The construction of the racial groups is based on empirical data from previous studies, which have highlighted that these groups are often subjected to discrimination and racialization in Sweden (e.g. Lange & Westin, 1997; Lundström, 2007; Kalonaityte et al., 2007; Sawyer, 2000)^.^^4^

In this study, I investigate attitudes towards interracial marriage with spouses representing the following groups: Adopted African (AA), Adopted Latin American (ALA), Adopted East Asian (AEA), African (A), Latin American (LA), Swedes (SWE) and South/East Asian (SEA).^5^ Rooth (2002) utilizes similar categories in his study of adoptees, based on the assumption of the visible differences associated with the countries of origin. Throughout the article, these groups refer to and include everybody who has their origin in these regions, regardless of if they were born in or outside of Sweden.^6^ This means that persons who have second and third generation migrant background are included in the categories. White Swedes in this study are defined as persons with two parents who have their origin in Sweden. I am fully aware of the issues and criticisms that arise concerning whether a geographical and national origin can define what is called a racial category and visible difference.

## Method and data

### Mixed methods

This study is a mixed-methods approach and based on some 461 postal survey responses and 28 semi-structured interviews conducted in the period November 2008 to October 2009. The postal survey aimed to measure attitudes, both directly and indirectly, by incorporating direct questions about interracial dating and marriage, and questions on interrelated issues such as attitude towards immigrants and immigration. Both the survey and interviews were carried out in Swedish. The Swedish words “blandäktenskap” (mixed marriage) or “blandrelation” (mixed relationship) were applied to describe interracial marriages. Measuring attitudes by means of surveys naturally has its shortcomings. One of the main issues is the possible gap between how the respondents answer the questions and how they act in reality. This study does not aim to understand the individual process of attitude construction or to prove the relationship between attitudes and actual behavior. Rather, the focus is to reach an understanding of the current attitudes and opinions towards interracial marriages that are expressed.

The postal survey sample was randomly selected from the governmental address registry SPAR, and it consisted of 2,000 residents of Malmö municipality between the ages of 18 and 78. A total of 622 respondents responded to the survey. When comparing the original sample with the number of respondents to the questionnaire, the return rate was lower among men and individuals in the age category 18–29. However, this is not unique to this survey, as it is common in other postal surveys. Appendix 1 shows the comparison of the proportion of people in each social category among the total sample and the respondents. Among the 622 respondents, this article will only focus on the responses given by 461 respondents of white Swedish background. Appendix 2 shows the characteristics of 461 respondents. Among 461 respondents, 178 reported to be married or cohabitating, whereof 149 reported a relationship with a white Swede. Moreover, the article only analyzes the responses given to the questions on whether the respondents can imagine marrying interracially, and whether they believe interracial marriages are accepted in Swedish society.

A total of 30 informants were chosen for interviews from among those who answered the survey. Three of the interviewees directly communicated their willingness to participate, while the other 27 were selected from the survey respondents who agreed to be contacted for the follow-up interviews by systematic random sampling according to their gender and residential area.^7^ Only 28 of the interviews are analyzed as 2 of the interviewees did not provide any effective answers.^8^ Among the 28 interviews that are analyzed, 16 are women and 12 are men. The ages of the informants ranged from 21 to 71 years, and they had diverse occupational backgrounds, including students, highly-qualified professionals, and unskilled workers. Some were employment seekers at that time. The interviewees were all of Swedish origin, with the exception of four, who had at least one parent of West European, Central East European or Latin American origin. I have included them in the analysis since they identified themselves as part of the majority society; the manner in which they answered the interview questions did not differ from other interviewees of a white Swedish origin. I carried out eight interviews face-to-face and the rest via the telephone. The responses show no significant differences in the length, effectiveness or the content of the interviews whether the interview was conducted face-to-face or by telephone. For ethical reasons, the interviewees and the answers that the interviewees gave to the survey cannot be matched and compared.^9^ All the answers given during the interviews are treated anonymously and confidentially.

The interviewees were asked to react and respond to the survey results, rather than to articulate their personal thoughts about the issue. This was an intentional choice and an attempt to eliminate the effect of social desirability needs that can be experienced by informants. Besides, as Ehn (1996) contends, when people comment on actual societal questions, such as “immigration and immigrants”, they tend to give answers based on how the interviewees believe others act and think rather than what they themselves think. Ehn states that this enables an interpretation of the interview result that reflects a social construction of meaningful experiences and cultural identity, rather than simply being an interpretation of what the informants think and have experienced (1996, p. 137). Another reason for letting the interviewees respond to the survey results, rather than articulating their personal thoughts, was my position in the research. I have visible differences that signal that I structurally do not belong to the majority population. By visible I not only mean my Asian appearance but also my name and language skills. I do not share the ascribed characteristics of the majority population and what is considered to be the norm of Swedishness. The choice to not ask the interviewees if they can imagine intermarrying directly was a choice to minimize the Race of Interviewer Effect (RIE), that is, “the ‘adjustment’ that people make to their opinions and attitudes when questioned by an interviewer from another racial or ethnic group” (Gunaratnam, 2003).

## Results and analysis

### Survey results

**Fig. 1:**
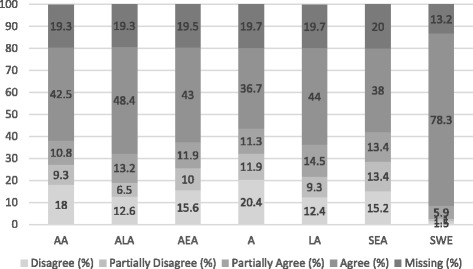
Can you imagine marrying someone of the following origin? Respondents of white Swedish Background (*N* = 416, %)

Figure 1 shows the 416 white Swedish respondents’ answers to the statement “I can imagine marrying someone of the following origin”. Missing cases (non-response) varies from 13% to 20%.^10^ The non-response could be due to respondents not having enough information to respond to the statements, refusing to respond to specific statements, or simply deciding not to respond to the statements; it may also be due to the sensitive nature of the statement. Those who did not respond were more often elderly, female respondents with a lower income and educational level. In addition, they tended to have no contact at all with different migrant groups and lived in residential areas with few migrants. This may indicate that respondents with higher incomes, educational levels, and more experience of interacting with diverse ethnic and racial groups are slightly more represented in the sample analyzed.

The descriptive statistics show that the majority of the respondents can imagine marrying interracially; however, there is a hierarchy of preference. The respondents could imagine marrying someone of Latin American, South/East Asian and African origin to a lesser extent than someone of Swedish origin. It should be noted that the majority of the respondents chose the alternatives “agree” or “disagree” and not the more neutral alternatives “partially agree” or “partially disagree”, which indicates a clear articulation of opinion. Among the three racial groups, Latin American received the most positive responses, while African received the least positive. Previous studies in Sweden show that the Latin American groups were perceived to be culturally closer to Swedes than South/East Asian and African (Lange & Westin, 1997; Mella & Palm, 2009; Mella & Palm, 2007). Therefore, that more respondents could imagine intermarrying with someone of Latin American origin than South/East Asian or African origin may be explained by the perception of cultural differences.

The dispersed preferences towards adoptees, namely the fact that Adopted Latin American was the most preferred group, followed by Adopted East Asian and Adopted African, should not be overlooked. This clearly shows that even though all adoptees are culturally Swedish, there is a hierarchy, depending on the origin and the visible differences of the adoptees that comes with the origin.

**Fig. 2:**
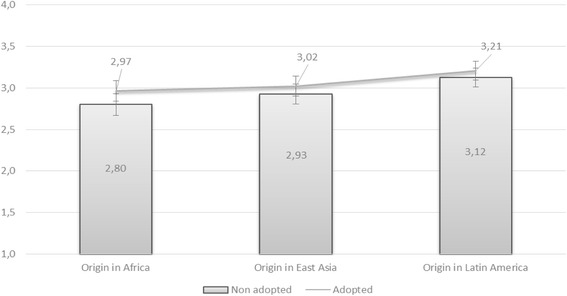
Attitudes towards interracial marriage: Comparison of white Swedish respondents’ attitudes towards non-white migrants and transnational adoptees in Sweden

Figure 2 presents the mean attitudes among the 461 survey respondents and shows 95% confidence interval. The closer the mean is to four, the more positive the response is.^11^ Even though the mean attitudes are slightly more positive for the adopted groups, the 95% confidence interval demonstrates that there are no statistically significant differences between attitudes towards the adopted groups and the equivalent migrant groups. In terms of cultural preference when selecting a marriage partner, the result is perplexing, considering adoptees grow up most often in a Swedish family, with a Swedish name, and Swedish language and culture. In this respect, an assumption can be made that the Swedish culture of the adoptees does not play as great a role as expected in the question of intermarriage and that attention should be cast on the role of visible differences. The assumed unimportance of the adoptees’ cultural backgrounds also explains the hierarchical preference between the three adopted groups, with Latin American adoptees receiving more positive responses and African adoptees receiving least positive responses among the three.

**Fig. 3:**
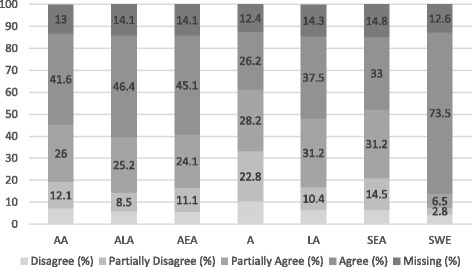
Is it accepted in Swedish society to intermarry? Respondents of white Swedish Background (*N* = 416, %)

Figure 3 shows the descriptive statistics to the responses given on whether respondents believed that marriages with different racial and adopted groups are accepted in Swedish society. While the previous question captures attitudes towards interracial marriages, this question captures the social norms of interracial marriages. Contrary to the previously presented result that shows almost no difference in attitudes towards non-white adoptees and equivalent migrant groups, it becomes clear that the respondents believe that adoptees, especially adopted Africans, are more socially accepted as marriage partners than someone of migrant origin.

**Fig. 4:**
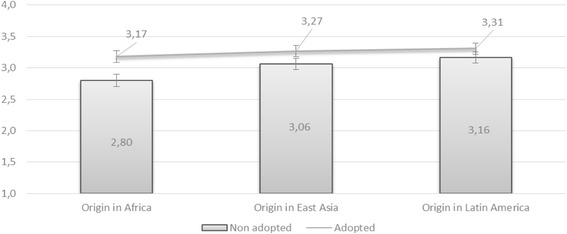
Social acceptance of interracial marriages: Comparison of white Swedish respondents’ attitudes towards non-white migrants and transnational adoptees in Sweden

Figure 4 illustrates the mean attitudes together with 95% confidence interval. Observing the 95% confidence interval, there is a statistically significant difference in the answers that were given towards adopted and migrant groups. Here, it can be argued that the perception of culture plays a role when it comes to respondents’ ideas regarding the acceptability of such unions in Swedish society. It can be reasoned that since adoptees are culturally Swedish, the respondents believed that it is more socially accepted to marry adoptees rather than someone of migrant origin. This can also be understood in terms of social desirability needs. Because the awareness that transnational adoptees are Swedish is strong in Sweden, survey respondents might have agreed that they are socially accepted as marriage partners.

The next section addresses the question of why adoptees are not as preferable as Swedes as marriage partners, even though the respondents believe that such unions are more socially accepted in comparison to unions between Swedes and someone of a migrant origin. The relationship between visible differences and the perception of cultural differences are explored through qualitative material.

## Interview results

### The idea of culture

The argument “It is the culture that matters” was the most frequent message that came across throughout the interviews in explaining the attitudes towards interracial marriage. For example, an interviewee stated that it was the “cultural difference [between the couple] that creates problems” and that attitudes towards interracial relationship had “nothing to do with racism”. Interviewees’ idea of culture inferred something different from “Swedish” and “Western” culture, and something that was a problem and incompatible with what they considered “Swedish culture”. The focus on cultural differences and the notion that “culture is something that is different from Swedish or Western culture” was prominent, especially when the interviewees expressed that some intermarriages were not as problematic as others, such as a marriage between a Swede and a Dane. This understanding of intermarriage being problematic if the spouses are of non-Western background corresponds with previous ethnographic studies carried out in Sweden (e.g. Gerholm, 2003; Begovic, 2003).

The interview materials show that the perception of culture and cultural similarities affect the attitudes towards interracial marriages. While Latin Americans were regarded as having cultural similarities, the focus was on cultural differences when the interviewees explained the attitudes towards marriages with the groups South/East Asian and African. One interviewee justified the more positive attitudes towards Latin Americans, compared to South/East Asians and Africans, as “[t]he closer it is to your own culture, the easier it is to accept”. Some interviewees articulated that Latin Americans are “originally European”. The informants linked the idea of culture to the notion of “origin” and repeatedly referred to geographical countries or areas: “there” rather than “here”. Based on the notion that cultures belong “there”, not “here”, the idea of culture is fixed and essentialized together with the idea of origin.

The idea of gender equality was incorporated into the idea of Swedish culture. Gender equality was considered as something that other “cultures” do not necessarily share. For example, one interviewee opined, “… maybe it is perceived that other cultures are more male-dominated. … I still believe that Sweden, the Swedish culture, is pretty equal; and gender equality is something important.” Nordic scholars have argued that the gender equality discourse is central to the national self-image of the Nordic countries (e.g. Hübinette & Lundström, 2011; Keskinen, 2009). Mulinari (2008), in particular, asserts that gender equality has developed in Sweden “as the central ethnic signifier of national belonging and the most important boundary between ‘us’ and ‘them’” (Mulinari, 2008, p. 180). A dichotomy between “patriarchal migrant cultures” and the “gender equal Swedish culture”, and the exclusion of non-Western men and women from the notion of gender equality can be observed in the explanations provided by the interviewees on attitudes towards interracial marriage. People who are perceived to have a “different culture” are ascribed as not sharing Swedish views on gender equality.

Some interviewees did express hierarchical attitudes when referring to visible differences, stating “the ones who have lighter skin color are more accepted” or “it maybe because they differ so much in appearance.” However, the perception of a different culture and gender equality value were given as the main reasons to the question why survey respondents could imagine marrying someone of Latin American, South/East Asian and African origin to a lesser extent than someone of a Swedish origin. This reasoning cannot be used to explain why adoptees are not preferred as much as Swedes as marriage partners, and why there is hierarchy among the adoptees, since adoptees are generally raised in a Swedish family, sharing Swedish cultural and speaking Swedish as their mother tongue. The next section moves on to discuss the interviewees’ reasoning regarding attitudes towards interracial marriages with non-white transnational adoptees, and it sheds light on the relevance of race and visible differences.

### Talking about visible differences

Interviewees who repeatedly and doggedly argued that the negative attitudes towards interracial marriage is due to the cultural and social differences seemed honestly shocked when presented with survey results that show insignificant differences in attitudes towards non-white adoptees and migrants. For example, one interviewee said, “… it actually doesn’t matter whether you are adopted, when you look at this result. Then you may say that my explanation about the cultural clash is not valid because it is purely racial if you look at the groups.”

The informants all shared the opinion that “if you are an adoptee, you are a Swede”. The majority of the interviewees could not find the words to explain why adoptees were not preferred as marriage partners as much as Swedes, or why there was so little differences between attitudes towards adoptees and migrants of the shared origins. The idea of colorblindness was persuasive: The informants all shared the opinion that “if you are an adoptee, you are a Swede”. A little less than two thirds of the interviewees articulated their confusion about the result and did not offer any further explanations. Eight informants specifically mentioned and insisted that adoptees were Swedes. Six interviewees could not offer anything other than “I really don’t know why” and “it’s strange”. This shows the prominence of color-blindness in Sweden and how Swedes are not used to talking about racial differences.

Some interviewees articulated that interracial marriage with adoptees would be “easier” than with someone of migrant background due to the lack of cultural differences. The remark that it is easier to have a relationship with an adoptee is interesting. While the comment indicates that adoptees’ cultural similarity with Swedes makes the relationship “easier”, compared to having a relationship with someone of migrant origin, it also infers that there is something about adoptees that makes a relationship with them somehow different to a relationship with white Swedes. I argue this not only because of the comparison that it is “easier” but also because none of the interviewees specifically articulated that having a relationship with adoptees is the same as having a relationship with someone of a Swedish origin, even though interviewees were convinced that “adoptees are Swedes”. What the previous quote and several others below express is that these differences infer the racial and visible differences. While looking at the survey results, one interviewee stated that the attitudes toward adoptees “then only have to do with the physical, like skin color and appearance, hair color, whatever it might be.” Another interviewee determinedly expressed the following in response to the non-existing differences in attitudes towards adoptees and migrants:

If you get closer to this person, get to know the person and understand that this person has another cultural background [than the appearance suggests] because he or she has grown up in the Swedish culture, then I think that the differences [in attitudes] should be bigger than shown here. But obviously the physical appearance remains; adoptees will still be judged by what they look like and where they were born, and so on. I think it is scary, and I cannot understand this really.

This quote indicates how racial groups may be constructed in Swedish society based on visible differences. Moreover, the interviewee’s words are a clear articulation of the existing norm of color-blindness in Sweden and an undeniable realization that race and visible differences matter there.

A couple of interviewees were, in fact, more daring and expressed the attitudes towards adoptees as “playing the safe card”. With the idea that adoptees share the same cultural values as the majority of Swedes, the interviewees stated that they do not know whether a person is adopted or not by his or her appearance. Since the survey questioned directly whether respondents can imagine marrying an adoptee, the “playing the safe card” argument is not sufficient to explain the survey results themselves. However, this comment depicts an interesting relationship between the idea of culture and visible differences, and how cultural differences may be inferred by the visible differences. It corresponds with previous studies in Sweden that indicate that culture is perceived according to how visibly different “they” are from “us” (e.g. Mattsson, 2005; Pred, 2000).

## Conclusion

Based on the survey results and interviews with white Swedish residing in Malmö, Sweden, this article focused particularly on the question of how race and visible differences of the imagined partner plays a role by comparing attitudes towards having spouses of migrant origin and non-white adoptees. Since non-white adoptees grow up in Sweden as culturally Swedish, and the only thing that divides them from the majority Swedish society is their racial background and visible differences, the comparison highlights the question of when culture and visible differences matter in the question of attitudes towards interracial marriage.

The survey results show that the majority of the respondents could imagine marrying someone of Latin American, South/East Asian and African origin, though to a lesser extent than someone of Swedish origin. The notion of culture was predominantly utilized by the interviewees to explain these hierarchical attitudes towards interracial marriages. The idea of cultural differences between the couple may explain why survey respondents could imagine marrying someone of Swedish origin the most and someone of Latin American, South/East Asian and African origin the least. However, the survey results indicate that the attitudes towards interracial marriage cannot solely be explained through the idea of cultural differences. The results revealed that non-white transnational adoptees, who are culturally Swedish but racially and visibly different from the majority Swedes, are not as preferred as marriage partners compared to white Swedes and that there are no statistically significant differences in attitudes towards non-white adoptees and non-white migrant groups. Furthermore, the dispersed preferences towards adoptees, namely Adopted Latin Americans as the most preferred followed by Adopted East Asians and Adopted Africans, indicates a hierarchy of preference according to the visible differences. The idea of cultural differences cannot explain the attitudes towards transnational adoptees or the dispersed preferences towards the three adopted groups. These results strongly indicate that race and visible differences matter in the question of choosing a marriage partner in Sweden. Despite the result that adoptees were not as preferred as marriage partners, the survey respondents believed that interracial marriages with non-white adoptees are more socially accepted than marriages with non-white migrants. The Swedish cultural background of non-white adoptees seemed to be recognized in the question of whether interracial marriages are socially accepted in Sweden.

When interviewees were faced with the survey results, most of them had difficulties talking about the racial and visible differences of the adoptees, although several interviewees specifically expressed that race and visible differences of the adoptee may affect the attitudes. Moreover, interview materials exhibited indications that visible differences may affect the perception of cultural differences. Interview results clearly showed the norm of color-blindness in Sweden and the difficulties of talking about race and visible differences.

This study focused solely on the attitudes towards interracial marriages; and the results clearly demonstrate that race and visible differences do matter in Sweden. The survey and interview results clearly show that even though transnational adoptees are more socially accepted as marriage partners, because of their cultural and linguistic compatibility, they are not as preferable compared to Swedes, because of their racial and visible differences. Combining the survey and the interviews, the complex and intricate reasoning behind the attitudes toward interracial relationships came to the surface. The results open up a discussion, and they question the idea of colorblindness in Sweden. I would like to stress the importance of including adoptees in studies of international migration and ethnic relations in order to shed light on how race and visible differences matter in the Swedish context. The comparison of non-white adoptees and persons of migrant origin can contribute to a deeper understanding of racism and discrimination – something that the idea of culture and ethnic differences cannot explain.

## Appendix 1

**Table 1:** Descriptive characteristics of the sample compared to the whole of Malmö Municipality (%)

		Sample (*N* = 620)	Malmö Municipality (*N* = 280.801) (Age 18–78)
Gender			
Male		46.3	49.0 (49.6)
Female		53.4	51.0 (50.4)
Age			
18–29		22.7	20.2 (26.3)
30–44		31.3	23.1 (30.2)
45–64		28.9	23.0 (30.1)
65–78		15.0	9.8 (12.8)
Population with immigrant background[1]		33.2	37
Population born outside Sweden		27.9	28
Top ten immigrant groups		Denmark	Denmark
		Poland	Former Yugoslavia
		Iraq	Iraq
		Bosnia and Herzegovina	Poland
		Finland	Bosnia and Herzegovina
		Turkey	Lebanon
		Germany	Iran
		Hungary	Hungary
		Macedonia	Germany
		Bosnia	Finland
Education at higher level [2](20-64)		45.6	40.0
Political Preference[3]			
Alliance (center-right)[4]		44.3	43.8
Red-Greens (social democratic-left)[5]		45.4	46.8

[1] The percentage for the sample shows the percentage of those who have reported to have at least one parent with an immigrant background or who being adopted from other countries, while the percentage for the municipality shows the percentage of those who were born outside Sweden or have two parents who were born outside Sweden.

[2] The percentage for the sample shows the percentage of those who have reported to have completed above secondary education (college and university), while for the municipality it is the percentage of those who have one or more year of college or university education at undergraduate and graduate level.

[3] Based on the response to the question of which party respondents voted for in the national election in 2006. Information relating to the municipality was taken from the election report published by the municipality, also based on the national election in 2006. Malmöstad, Valet i Malmö 2006 (Election in Malmö 2006). The percentage for the sample eliminates 94 individuals who answered that they did not vote.

[4] Consists of the Moderate Party, the Center Party, the Liberal People’s Party and the Christian Democrats.

[5] Consists of the Social Democratic Party, the Left Party and the Green Party.

## Appendix 2

**Table 2:** Comparison of background characteristics of the white European respondents analyzed in logistic regression

		Total sample of white European background (*N* = 461)	Total Sample (*N* = 620)
Gender (%)	Male	47.3	46.3
	Female	52.5	53.4
Age (%)	18–44	50.8	54.0
	45–64	29.3	28.9
	65–78	17.8	15.0
Education at higher level (%)		46.9	42.7
Political preference (%)	Alliance (center-right)	48.5	44.3
	Red-Greens (social democratic-left)	40.2	45.4

## Footnotes

1 Throughout the article the terms intermarriage and interracial marriage will be used to refer to a legal marriage but also to cohabitation between a person of Swedish origin and a person of migrant origin or a non-white adoptee. The results presented in this article is based on my doctoral dissertation (Osanami Törngren, 2011)

2 In this study respondents of white Swedish background are respondents who reported that neither of their parents originated from a country other than Sweden nor were adopted (*N* = 404), and those who reported an origin in Scandinavia (*N* = 31), Southern Europe (*N* = 6), Western Europe (*N* = 14) including North America, and respondents who reported a mixture of the previously named origins (*N* = 6).

3 See for example Hübinette and Lundström (2011) and Hübinette and Tigervall (2009) for more detailed discussion.

4 See Osanami Törngren (2011) for more detailed discussion.

5 In the survey the *African* are defined as those who have their origin in for example Ethiopia, Ghana, Tunisia and Gambia, *Latin American* as those who have their origin in for example Chile, Uruguay, Argentina and Mexico, and *South/East Asian* as those who have their origin in for example Vietnam, Thailand, China and the Philippines (SEA).

6 In the original study attitudes towards Central East European, Middle Easterner, Scandinavian, South European and West European are included as well. See Osanami Törngren (2011).

7 The interview content from the three interviewees who volunteered did not show any indication of deviant attitudes or expression, neither positive or negative, compared to the other randomly selected interviewees. A total of 194 people were first of all systematically categorized according to gender and place of residence. After this systematic categorization the interviewees were randomly selected. Initial contact was made via the telephone; all except one accepted their participation in interviews. The person who refused to participate in the interview was a Danish woman working in Denmark and Malmo was only a place of residence and she was not interested in how Sweden dealt with its integration politics.

8 The two interviews that were eliminated only contained answers such as “yes”, “no”, “I don’t know” which were not possible to analyze deeper.

9 The ethics code of conduct in Swede does not allow the interviewee to know the individuals answers that the interviewees gave to the survey.

10 See Osanami Törngren (2011) for more detailed discussion on missing cases.

11 Responses are coded agree (4), partially agree (3), partially disagree (2) and disagree (1).
